# Reversing a global health workforce crisis

**DOI:** 10.2471/BLT.14.151209

**Published:** 2015-01-01

**Authors:** Michel Sidibé, James Campbell

**Affiliations:** a Joint United Nations Programme on HIV/AIDS, Geneva, Switzerland.; b Global Health Workforce Alliance, World Health Organization, avenue Appia 20, 1211 Geneva 27, Switzerland.

The current Ebola virus outbreak in western Africa has exposed vulnerable health systems, dire shortages of health workers and a deep mistrust between authorities, health workers and the communities at risk. Policy-makers responsible for health systems need to investigate what is not working and what can be done to make systems resilient, sustainable and, ultimately, ready to meet the challenges of the next global pandemic.

Shortcomings in the health workforce stretch well beyond Africa and current disease outbreaks. The Open Working Group on Sustainable Development Goals proposed a broad health agenda – the ambitions of which are yet to be matched by investment in the health workforce.^1^ We simply need more health workers. In 2013, to reach a threshold of just 34.5 skilled health professionals per 10 000 population, approximately 7.2 million more midwives, nurses and physicians were needed – and this shortfall is predicted to rise to at least 12.9 million in the coming decades.^2^

Our current, outdated model of human resources for health needs an urgent upgrade. In country after country, we are seeing that top-down, facility-based, doctor-dependent, disease-focused models of health care are neither ideal nor sustainable. We need a more balanced workforce that is tailored to each country’s needs. Education, training and incentives should be focused on creating an efficient workforce that is centred on people rather than disease.

In health systems, quality can be assured with task-shifting but success in task-shifting is contingent upon having the correct mix of skills, supervision and support structures in place.^3^^–^^5^ In the treatment of human immunodeficiency virus (HIV) infection, uptake and outcomes have been improved by redeploying health workers and enabling community-based practitioners to serve vulnerable communities.^6^

Fragmented or parallel services are improved when provided in a more patient-centered and integrated way.^7^ For example, health outcomes are rapidly improving in Rwanda, a country that has taken a systems-strengthening approach in which community health workers are the core of the health system.^8^

The post-2015 development agenda needs to address many major issues, but few are more pressing than the global health workforce crisis. The Global Health Workforce Alliance is leading development of a global strategy on human resources for health that will promote an integrated approach to workforce development.^9^ This strategy will need to achieve four main elements. The first element is a human resource model that is fit-for-purpose. Such a model recalibrates workforce composition as a function of local risk and disease burden profiles in target communities. The second element is to strengthen national governance and coordination frameworks. Governance of the health workforce must be multisectoral, with stakeholders from health, finance, education, labour and social care ministries, labour unions and the private sector. The third element is to scale up “smart” spending. New approaches to investing in the health workforce should be reflected by national business plans. Spending should be responsive to national needs and supported by greater alignment across the donor community. To address the trans-border dynamics, fragmentation, gaps and inefficiencies that hinder national solutions, the *Global code of practice on the international recruitment of health personnel*^10^ should be rigorously enforced. Finally, as no goals for health will be achieved without a strong health workforce, the strategy will need political commitment from multisectoral bodies, such as the G20, G7 and regional blocs.

The Ebola virus outbreak has demonstrated the perils of not investing in human resources and other components of health systems. Many global leaders will have an opportunity to inform the post-2015 agenda when they next meet as the Executive Board of the World Health Assembly. Shared investment in a strong health workforce is needed now, so that we can face the next pandemic with resilience rather than fragility, with coordinated action rather than fragmentation and with confidence instead of fear.
